# A Wide-Range and Calibration-Free Spectrometer Which Combines Wavelength Modulation and Direct Absorption Spectroscopy with Cavity Ringdown Spectroscopy

**DOI:** 10.3390/s20030585

**Published:** 2020-01-21

**Authors:** Zhen Wang, Yanjun Du, Yanjun Ding, Zhimin Peng

**Affiliations:** State Key Laboratory of Power Systems, Department of Energy and Power Engineering, Tsinghua University, Beijing 100084, China; wangzhen16@mails.tsinghua.edu.cn (Z.W.); duyanjun@mail.tsinghua.edu.cn (Y.D.); dyj@tsinghua.edu.cn (Y.D.)

**Keywords:** continuous wave cavity ringdown spectroscopy, wavelength modulation and direct absorption spectroscopy, CO, calibration free, wide range

## Abstract

A wide-range, calibration-free tunable diode laser spectrometer is established by combining wavelength modulation and direct absorption spectroscopy (WM-DAS) with continuous wave cavity ringdown spectroscopy (CW-CRDS). This spectrometer combines the benefits of absolute concentration measurements, wide range, and high speed, using WM-DAS with enhanced noise reduction in CW-CRDS. The accurate baseline ringdown time, *τ*_0_, is calculated by the absorption peak (measured by WM-DAS) and the ringdown time containing gas absorption information (measured by CW-CRDS at the center wavelength of the spectral line). The gas concentration is obtained without measuring *τ*_0_ in real time, thus, greatly improving the measuring speed. A WM-DAS/CW-CRDS spectrometer at 1.57 μm for CO detection was assembled for experimental validation of the multiplexing scheme over a concentration ranging from 4 ppm to 1.09% (0.1 MPa, 298 K). The measured concentration of CO at 6374.406 cm^−1^ shows that the dynamic range of this tunable diode laser absorption spectrometer is extendable up to five orders of magnitude and the corresponding precision is improved. The measurement speed of this spectrometer can extend up to 10 ms, and the detection limit can reach 35 ppb within 25 s.

## 1. Introduction

Tunable laser diode absorption spectroscopy (TDLAS) [1,2,3] has the advantages of being noncontact, fast response, and wavelength selective. The direct absorption spectroscopy (DAS) [4,5,6] in TDLAS has a clear physical concept and is easy to operate, which is frequently used to quantify concentration, temperature, and other parameters of gases. DAS has also been widely used in the case of high concentrations and strong absorption environments (absorbance 0.01 to ~1). For trace gas monitoring or weak absorption line measurements (absorbance <10^−3^), techniques with a higher signal-to-noise ratio (SNR) are used, such as cavity-enhanced absorption spectroscopy (CEAS) [7,8,9] or continuous wave cavity ringdown spectroscopy (CW-CRDS) [10,11,12].

In some environments, the gas concentration can change from a trace to a high level in a matter of minutes and occasionally fluctuates violently. For example, when monitoring the concentration of water in the upper atmosphere on a moving aircraft, it can range from ~1 ppm to 1% and fluctuate sharply as the aircraft passes through the clouds [13]. In the atmospheric, laminar and non-premixed CH_4_/air model flames, the concentration of CO at different heights in the flame ranges from ~1 ppm to 4% [14]. The concentration of engine exhaust gases (e.g., NO and CO) in a single internal combustion engine cycle can range from ~1 ppm to 0.3% (NO) and ~1 ppm to 4% (CO) [15]. There is also quite a large difference in the radial profile of the HO_2_ concentrations throughout the plasma effluent in an atmospheric pressure plasma jet [16]. In these cases, such a wide range single method (DAS, CW-CRDS, or CEAS) for fast measurements of gas concentration is difficult to achieve. Because of the weak signal and the short ringdown time at high concentrations, CW-CRDS requires higher gain detectors and faster data sampling rates [17,18,19]. Likewise, owing to the weak gas absorption at low concentrations, DAS needs a longer optical path [20] or stronger mid-infrared gas absorption lines which further require mid-infrared quantum cascade lasers [21] or interband cascade lasers [22]. One of the simplest solutions is to combine various methods, such as the DAS and wavelength modulation spectroscopy (WMS) [23,24,25]. By using the quantitative results of DAS to calibrate the WMS, the online measurement of water concentration over a wide range and with high precision can be realized [26]. The combination of CW-CRDS and laser-induced fluorescence (LIF) facilitates the real-time and high-precision measurement of NO*_x_* in the atmosphere [27]. The complementary nature of CEAS and CW-CRDS methods also facilitates the real-time and high-precision detection of N_2_O_5_ in the atmosphere [28]. Therefore, this paper focusses on the combination of CW-CRDS and DAS. Simultaneously, the wavelength modulation and direct absorption spectroscopy (WM-DAS) [29,30,31] method, which is based on sinusoidal modulation and fast Fourier transform (FFT) analysis, proposed by our group recently, further improves the accuracy of DAS. With this method, the root mean square error (RMSE) of spectral fitting reaches ~3 × 10^−5^ [30] and, then, the detection limit of WM-DAS can reach ~3 × 10^−9^ cm^−1^ which is realized by using a long optical path cell of ~100 m (e.g., Herriott cell [32]). In this way, WM-DAS obtains a large intersection with the measurement range of CW-CRDS. Thus, a wide range of measurement is easily obtained by the combination of WM-DAS and CRDS. This large intersection area between the two methods makes it possible to obtain the calibration-free baseline ringdown time.

This paper describes a simple method based on WM-DAS and CW-CRDS to construct a wide-range and calibration-free spectrometer for measuring gas concentration. The measuring range and accuracy of the proposed spectrometer were analyzed theoretically and verified by measuring CO (6374.406 cm^−1^) in various concentrations at room temperature and normal atmospheric pressure. Merging the benefits of WM-DAS (fast response and calibration free) with CW-CRDS (high precision), a wide measuring range and a large intersection area between the two methods are obtained. In this intersection area, the accurate baseline ringdown time, *τ*_0_, is calculated by using the peak absorbance (measured by WM-DAS) and the ringdown time containing gas absorption information (measured by CW-CRDS at the center wavelength of the spectral line). In this fashion, free calibration of *τ*_0_ is realized and the connection between the two methods is established through *τ*_0_. Moreover, the measuring speed and the detection limit of this spectrometer are further improved.

## 2. Experimental Systems

**Figure 1:**
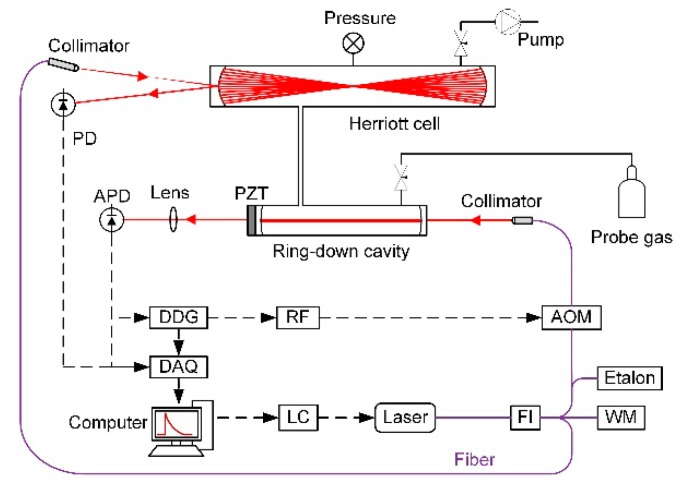
System schematics for the continuous wave cavity ringdown spectroscopy (CW-CRDS) and wavelength modulation and direct absorption spectroscopy (WM-DAS) methods. LC, laser current and temperature controller; FI, fiber isolator; AOM, acousto-optic modulator; PD, photodiode; APD, avalanche photodiode; DDG, digital delay generator; PZT, piezoelectric transducer; WM, wavelength meter; RF, radiofrequency; and DAQ, data acquisition.

The system schematics for CW-CRDS [10,11,12] and WM-DAS [29,30] are shown in Figure 1. The light source, in both the cases, is a DFB laser which has a continuous wavelength ranging from 1566 to ~1570 nm (6369 to ~6386 cm^−1^). The laser was tuned by changing the temperature and the current. The laser beam propagated through an optical isolator to reduce the optical feedback to the diode laser and, then, divided into four beams. One of the beams was focused on an acousto-optic modulator to generate the first-order beam that was, subsequently, focused into the ringdown cavity. The other three beams entered the Herriott cell [32], the wavelength meter, and the etalon, respectively. The free spectral region of the etalon is 1.5 GHz (~0.05 cm^−1^ @ 6381 cm^−1^) and the measurement accuracy of the wavelength meter is 0.2 ppm (0.0013 cm^−1^ @ 6381 cm^−1^).

The optical cavity (length, 50 cm) was formed by a pair of high-reflectivity (curvature, 1 m and R > 0.999975 at 1530 nm) mirrors. The piezoelectric transducer (PZT) was used to scan the cavity length. The magnitude and rate at which the cavity was modulated were determined by the desired quality of the resulting spectrum. Light was collected after the second cavity mirror using a lens and an InGaAs avalanche photodetector (Thorlabs, Inc., Newton, USA). When the buildup reached the preset trigger level of 1.5 V, the digital delay generator (DDG) sent out a pulse to the radiofrequency (RF) source that de-energized the acousto-optic modulator (AOM); thus, shutting off the light from going into the cavity. The data acquisition (DAQ) card, then, simultaneously collected the pulse signals and the ringdown signals and processed them using LabVIEW software in real time. The sampling rate was 20 MHz and the ringdown event sampling duration was 200 μs. The data analysis program ascertained the position of the rising edge of the pulse signal as the starting point and removed the initial 0.2 μs from the top of the ringdown curve while retaining the rest of the signal. The system used a fast fitting algorithm [33] to calculate the ringdown time measured at about 100 Hz.

The Herriott cell was formed by a pair of flat-concave mirrors. The distance between the two mirrors was 1.05 m, and the effective optical path was about 120 m. The emergent light was collected using an InGaAs photodetector, and the signals from the photodetector were transmitted to the DAQ card and, simultaneously, processed by the LabVIEW program. The relative laser wavelength was measured by the etalon.

## 3. Methods

### 3.1. WM-DAS

On the basis of DAS [4,5,6], the WM-DAS uses high-frequency sinusoidal modulation and FFT to filter noises to precisely recover the absorption spectrum [29]. In this technique, the laser wavelength is scanned periodically by modulating the laser current by a sinusoidal signal, and the laser is received by the detector after passing through the gas absorption cell. The light intensity of the laser can be defined by the following formula:

(1) $$I\left(x\right)=\sum_{k=0}^{\infty }A_{k}\cos\left[k\left(\arccos x-\eta \right)\right]\pm B_{k}\sin\left[k\left(\arccos x-\eta \right)\right]$$

where *k* = 0, 1, 2, …, −1 ≤ *x* ≤ 1, *A* and *B* are the real and imaginary parts of the Fourier coefficients of the characteristic frequencies, and *η* is the initial phase angle. The relative wavelength of the laser can be defined by the following formula:

(2) $$\nu \left(x\right)=\nu_{0}+a_{1}x+a_{2}\left[\left(2x^{2}-1\right)\cos\varphi_{2}\pm 2x\sin\varphi_{2}\sqrt{1-x^{2}}\right]$$

where *v*_0_ is the laser center wavelength. In Equations (1) and (2), “−” and “+” are used in the left (*V*1*V*2) and right (*V*1*V*3) periods, respectively [29] The coefficients (*a*_1_, *a*_2_, *η*, and *φ*_2_) can be obtained by measuring the relative wavelengths of the laser using an etalon. Through the above formula, the transmitted light intensity (*I*_t_) with gas absorption and the transmitted light intensity (*I*_0_) without gas absorption can be obtained, and the absorbance, *α*(*v*), can be obtained from the Beer–Lambert’s law:

(3) $$\alpha \left(v\right)=-\ln\left[\frac{I_{t}\left(\nu \right)}{I_{0}\left(\nu \right)}\right]=PS\left(T\right)XL\varphi \left(v\right)$$

where *P* is pressure, *S* is the line intensity, *T* is temperature, *X* is the mole fraction of the gas under study, *L* is the optical path, and *φ*(*v*) is the line shape function. At atmospheric pressure, *φ*(*v*) can be described by the Voigt line shape [34].

**Figure 2:**
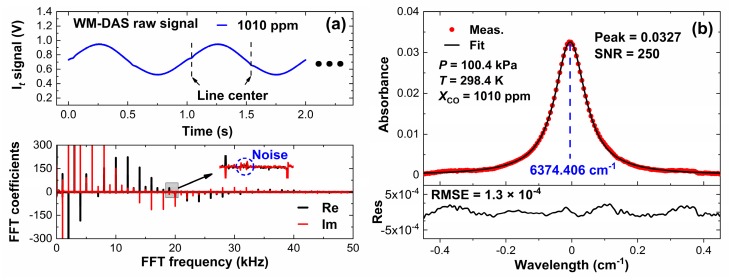
(**a**) Original WM-DAS signal and the corresponding fast Fourier transform (FFT) spectrum and (**b**) recovered absorbance of CO.

Figure 2a,b shows the originally transmitted signals and the recovered spectrum at 6374.406 cm^−1^ for a CO concentration of 1010 ppm. As shown in Figure 2a, only the harmonics of characteristic frequencies are extracted to reconstruct the signal of the transmitted light, and the noises from other frequencies (~19.4 kHz) can be effortlessly removed. As shown in Figure 2b, even if the effective optical path is 120 m, the root mean square error (RMSE) of the spectrum is about 1.3 × 10^−4^ (2.72 × 10^−6^ cm^−1^) at room temperature and normal atmospheric pressure and is smaller than that reported in the literature [26], which proves that the WM-DAS method has high accuracy.

### 3.2. CW-CRDS

The CW-CRDS typically uses wavelength scanning or cavity length scanning to couple a laser mode with a cavity mode [10,11,12] (this paper uses cavity length scanning), then, the ringdown signal is collected and the corresponding ringdown time (*τ*) is calculated. The absorption coefficient (*κ*) related to *τ* is [17,18,19]:

(4) $$\kappa \left(v\right)=\frac{1}{c}\left(\frac{1}{\tau \left(\nu \right)}-\frac{1}{\tau_{0}\left(\nu \right)}\right)=PS\left(T\right)X\varphi \left(\nu \right)$$

where, *c* is the speed of light, *τ*_0_ is the ringdown time of the evacuated cavity which depends on the reflectivity *R* of the cavity mirrors and losses including absorption by the dielectric coating and scattering on the surfaces and interiors of the mirrors. Herein, *P* is pressure, *S* is the line intensity, *T* is temperature, *X* is the mole fraction of the probe gas, and *φ*(*v*) is the line profile. Since the (1 − *R*)/*L* term can be treated as the baseline, the absorption line profile can be attained by fitting the measured curve 1/*cτ*(*v*). Nonetheless, the measurement technique which scans the whole absorption line can take a long time in doing so, and hence is not suitable for online measurement. The measurement speed can be improved by fixing the laser wavelength at the center of the absorption line and measuring only the ringdown time at this wavelength. By fixing the laser wavelength, Equation (4) can be simplified to:

(5) $$\kappa_{\nu_{0}}=\frac{1}{c\tau }-\frac{1}{c\tau_{0}}$$

where *τ*_0_ and *τ* are the ringdown times of the perturbing and the probe gas, respectively. In this way, the gas concentration can be determined without scanning the whole spectral line and the only parameter that needs to be calibrated is the baseline ringdown time *τ*_0_.

**Figure 3:**
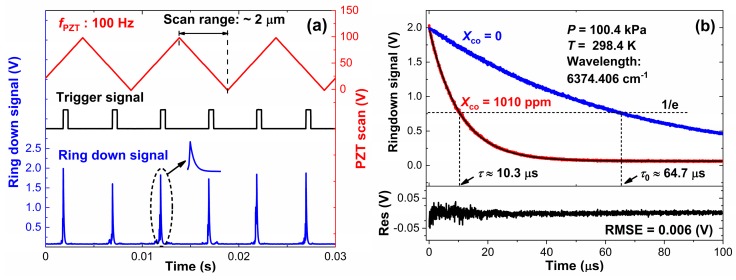
(**a**) Illustration of cavity length scanning, DDG trigger signal (black), light intensity (blue), PZT scan signal (red) and (**b**) ringdown signals of perturbing gas (blue) and probe gas (red) at 6374.406 cm^−1^.

Figure 3a shows the working principle of CW-CRDS using cavity length scanning at the center wavelength of the CO (6374.406 cm^−1^) spectrum. The cavity modes can be coupled to the laser wavelength by PZT scanning (red). In order to facilitate the fast acquisition of ringdown time, the amplitude and rate of PZT were set at about 2 μm and 100 Hz. When the emergent light (blue) of the cavity reaches the threshold voltage (~1.5 V), the DDG sends pulses (black) to turn off the AOM in order to obtain the ringdown signals. Figure 3b shows the ringdown signals of the perturbing gas (blue) and the probe gas (red), and their corresponding ringdown time are 10.3 μs and 64.7 μs, respectively. The absorption coefficient, as calculated by the Equation (5), is 2.72 × 10^−6^ cm^−1^, which equals the result of WM-DAS in Section 3.1.

### 3.3. Calibration-Free Baseline Ringdown Time

The above scheme, in Section 3.2, needs to measure the baseline ringdown time, *τ*_0_, which is not convenient enough for in situ measurements. Hence, a connection between the two (WM-DAS and CW-CRDS) gas chambers which have the same gas parameters are set up herein, following which, *α* = *L κ* can be obtained by combining Equation (3) with Equation (4). Thus, *τ*_0_ (at the center wavelength *v*_0_) can be obtained:

(6) $$\frac{\alpha_{\max}}{L}=\frac{1}{c\tau }-\frac{1}{c\tau_{0}}$$

where *α*_max_ is the absorbance peak of the probe gas measured by WM-DAS and *τ* is the ringdown time containing gas absorption information measured at the central frequency *v*_0_. According to Equation (6), the baseline ringdown time, *τ*_0_, can be calculated by *α*_max_ and *τ*. In this fashion, the baseline ringdown time, *τ*_0_, becomes calibration free and there is no need to take into account the small changes due to environmental factors. The measurement frequency of CW-CRDS can easily reach 100 Hz, which is similar to previous reports in the literature [17,18,19].

## 4. Analysis and Verification of the Measuring Range of the Proposed Spectrometer

**Figure 4:**
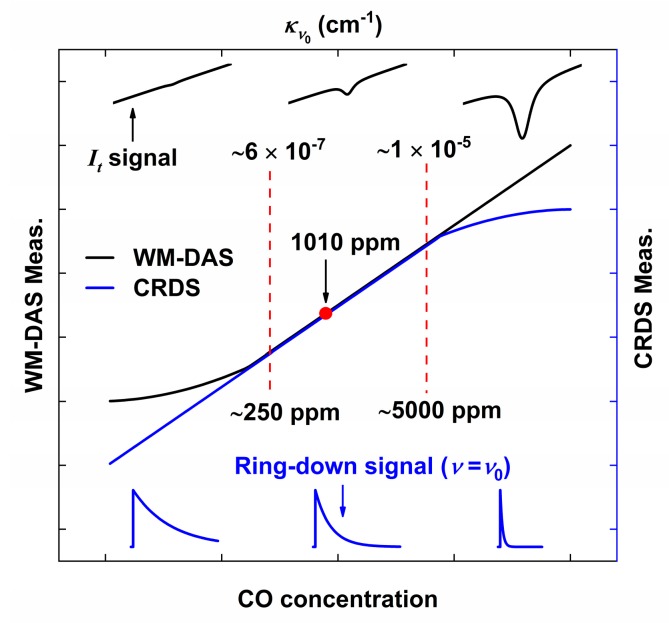
Relationships between ringdown signals at *v*_0_, emergent light signals, absorption coefficient and concentration of CO.

Figure 4 shows the range over which the CO concentrations were measured by the two methods (WM-DAS and CW-CRDS) at room temperature and normal atmospheric pressure. When the concentration is lower than 250 ppm, the absorption coefficient is found to be less than 6 × 10^−7^ cm^−1^ and the equivalent absorbance is less than 7.2 × 10^−3^. In keeping with the detection limit (7 × 10^−5^ [29,30,31], at room temperature and normal atmospheric pressure) of WM-DAS, the SNR is only ~103. When the concentration is more than 5000 ppm, the absorption coefficient is over 1 × 10^−5^ cm^−1^ and the SNR of WM-DAS with a 120 m Herriott cell lies close to ~2000. Strong absorption considerably weakens the emergent light from the ringdown cavity and reduces the ringdown time to less than 3.2 μs (the ringdown time of the evacuated cavity is 66.7 μs), which in turn, reduces the measurement accuracy of CW-CRDS. In order to circumvent this problem and improve the accuracy at high concentrations, CW-CRDS necessitates higher gain detectors and faster DAQ sampling rates. Likewise, at low concentrations, in order to improve the accuracy of WM-DAS measurements, it is necessary to consider a longer optical path (for instance a specially designed multiple reflection cell [35]) or to choose stronger mid-infrared gas absorption lines that require mid-infrared quantum cascade lasers [21] or interband cascade lasers [22]. Fortunately, the measuring range of WM-DAS with the 120 m Herriott cell has a large intersection with the CW-CRDS, and therefore the spectrometer established in this paper is the outcome of the process of combining the advantages of WM-DAS and CW-CRDS to achieve a single spectrometer having a wide and continuous range, as well as high precision for gas concentration measurement. Moreover, using Equation (6), the baseline ringdown time, *τ*_0_, can be calculated from the peak absorbance, *α*_max_, measured by WM-DAS and the ringdown time, *τ*, which contains gas absorption information as measured by CW-CRDS in the 250–5000 ppm range.

**Figure 5:**
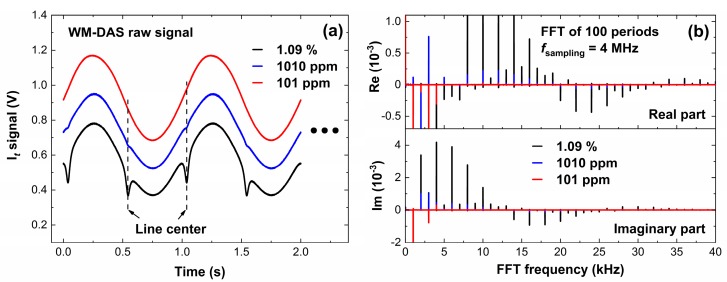
(**a**) Original signals of the transmitted light intensity at different concentrations measured by WM-DAS and (**b**) FFT spectrum of different concentrations.

**Figure 6:**
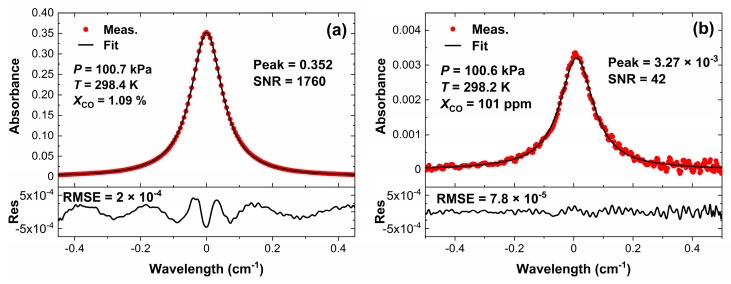
Restored absorbance at different concentrations of CO: (**a**) Concentration of 1.09% and (**b**) concentration of 101 ppm.

Two different concentrations of CO (1.09% and 101 ppm) were used to verify the relationships between ringdown signals at *v*_0_, emergent light signals, absorption coefficient, and concentration of CO, as shown in Figure 5. Figure 5a shows the transmitted light intensity of CO in different concentrations measured by WM-DAS, from which we can note that the absorption for 101 ppm is very weak and almost submerged by noise. However, the characteristic frequencies (1 kHz, 2 kHz, …) and noises can still be distinguished by the FFT spectrum as shown in Figure 5b. The absorbance of 1.09% is very strong and the non-zero light intensity at the central wavelength indicates that the WM-DAS can measure a higher concentration (about 5%). Figure 6a,b shows the recovery absorbance of 1.09% and 101 ppm, wherein the minimum value of RMSE can reach up to 7.8 × 10^−5^ at a peak absorbance of 0.32%. Due to the wide wavelength range (±0.45 cm^−1^) and the minor fluctuations of CO concentration at atmospheric pressure, the RMSE becomes slightly larger than that measured at lower pressures [30]. Therefore, the SNR in the spectrum of 1.09% is about 1760 less than the theoretical value shown in Figure 4. The experimental results show that the SNR in the spectrum of 101 ppm is ~42, and for 250 ppm it is ~104, which is consistent with Figure 4. In the range of 250 ppm to 1.09%, the SNR is suitable enough for gas concentration measurement, displaying the wide measurement range and high accuracy of WM-DAS.

**Figure 7:**
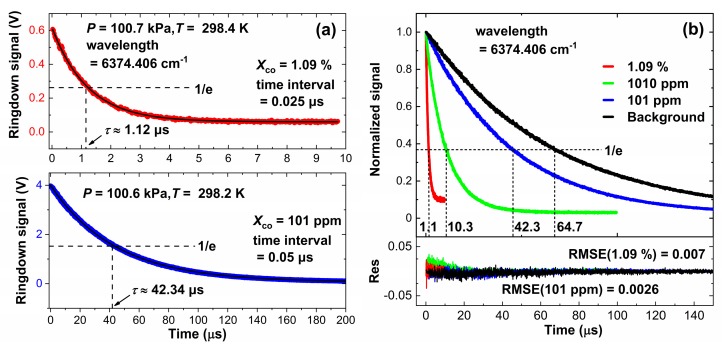
(**a**) Ringdown signals at different concentrations and (**b**) normalized ringdown signals and fitting residuals.

The experimental conditions for CW-CRDS are the same as those for WM-DAS. As shown in Figure 7a, the ringdown time is only 1.12 μs at high concentration (1.09%) and the maximum light intensity of the ringdown curve is only 0.6 V. At low concentration (101 ppm), the light intensity can reach up to 4 V and the ringdown time is approximately 43.9 μs. Figure 7b normalizes the ringdown curves in Figure 7a, where the RMSE at high concentration (1.09%) is much larger and is about four times that at low concentration (101 ppm). Therefore, it can be concluded that a strong absorption diminishes the light intensity and the sampling points on the ringdown curve, thus reducing the SNR, which is consistent with the conjecture of Figure 4. The ringdown time of the empty cavity is slightly less than 66.7 μs, which could be due to the slight presence of pollutants on the surface of the cavity mirror.

## 5. Results and Discussion of the Wide Range and Calibration-Free Technique

**Figure 8:**
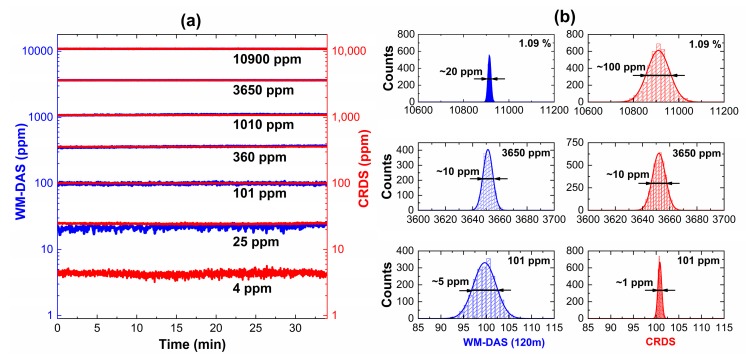
(**a**) The results of long-term measurement by WM-DAS and CW-CRDS at different concentrations and (**b**) histogram of the measured data and the best fits of Gaussian profile, and FWHM (equal to 2.355 times the standard deviation) of Gaussian profile.

Figure 8a shows the measuring range and accuracy of CW-CRDS and WM-DAS at different concentrations. For each method, the measurement over 30 min, revealed that the fluctuation of ambient temperature was less than 0.2 K, and the concentration of CO ranged from 4 ppm to 1.09%. The standard deviation corresponds to the measurement accuracy [26]. Allowing for the regional division in Figure 4, Figure 8b shows the typical 1.09%, 3650 ppm, and 101 ppm measurements, all of which show good Gaussian distributions. The two methods have the same measurement accuracy between 101 ppm and 3650 ppm. When the concentration is higher than 3650 ppm, the accuracy of WM-DAS becomes increasingly better. Conversely, when the concentration is less than 101 ppm, the accuracy of CW-CRDS is better. These experimental results further validate the inference from Figure 4.

**Figure 9:**
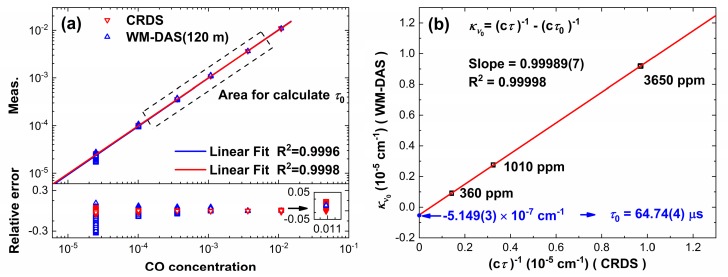
(**a**) Measurement results of CW-CRDS and WM-DAS and their relative errors with standard concentrations and (**b**) use of overlapping regions in the two methods to calculate *τ*_0_.

According to Equation (6), the baseline ringdown time, *τ*_0_, can be calculated directly without the need to measure the empty cavity. As shown in Figure 9a, the measurement accuracy of both methods is high in the 360–3650 ppm range, which implies their suitability for calculating *τ*_0_. Figure 9b displays the relationship between (*cτ*)^−1^ (measured by CW-CRDS at center wavelength) and *κ* (measured by WM-DAS, where *κ* = *α*/*L*). These data show a good degree of linearity of 0.99998 and the slope (0.99989) is almost equal to one, which is in good agreement with the Equation (6). In addition, *τ*_0_ can be obtained from the intercept (*cτ*_0_)^−1^ which can, in turn, be obtained by the linear fitting of these data. The calculated *τ*_0_ is 64.74(4) μs.

**Figure 10:**
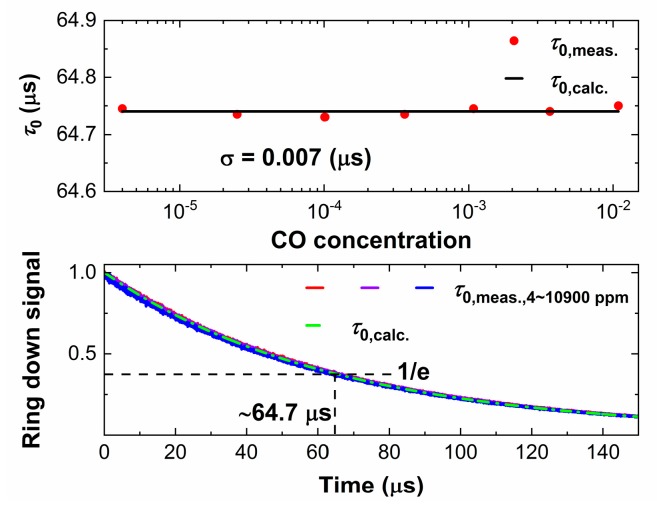
Measured and calculated values of *τ*_0_.

Figure 10 shows the calculated *τ*_0, calc_ and the measured *τ*_0, meas_. Before each measurement displayed in Figure 8a, the cavity was purged with nitrogen (purity 99.999%) for ~5 min, followed by the baseline ringdown time *τ*_0, meas_ measurement which was averaged after the intra-cavity pressure became stable. The standard deviation between the measured *τ*_0, meas_ and the calculated *τ*_0, calc_ values is about 0.007 μs (relative error is 1.08 × 10^−4^), which implies that the calculated value *τ*_0, calc_ is closer to the average value of *τ*_0, meas_.

Table 1 shows CO concentrations calculated by using *τ*_0, meas_ and *τ*_0, calc_, wherein the relative errors are less than 4 × 10^−4^. These results confirm that the gas concentration can be accurately measured by using *τ*_0, meas_. At high concentrations, due to the short ringdown time (*τ*_meas_) and the weak ringdown signal, the SNR is low and the error between the measured concentration (*X*_meas._ and *X*_calc._) and *X*_CO_ can reach up to ~10 ppm. It is worth noting that in real-time poor industrial field measurements, *τ*_0_ will attenuate slowly due to the pollution on the mirror surface [10,11,12,17,18,19]. However, following the present work, *τ*_0_ can be obtained by linear fitting of (*cτ*)^−1^ and *κ*, which is beneficial for the development of simple, fast, and calibration-free gas sensors.

**Figure 11:**
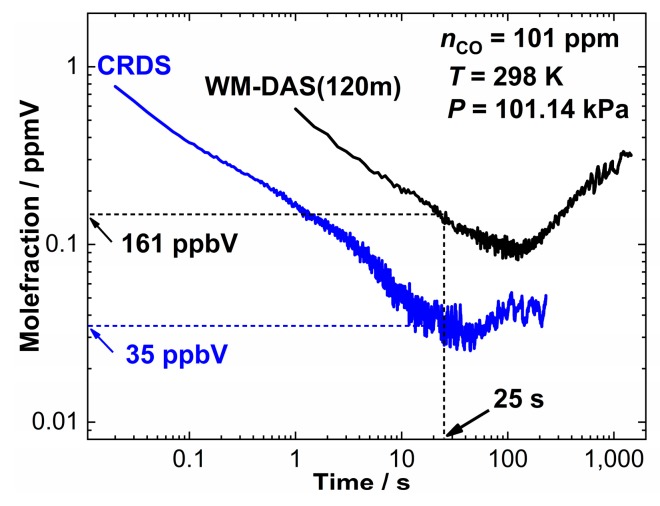
Allan standard deviation of two methods at 101 ppm.

To further compare the two spectroscopic techniques, a calculation of the Allan deviation [36] for a simultaneously measured concentration of 101 ppm was conducted under identical conditions (Figure 11). At the integration time of 25 s, the detection limit of CW-CRDS reaches 35 ppb which is equivalent to that found in the literature [37,38] and for WM-DAS it is 161 ppb which is comparable to WMS [39,40] and is five times greater than that of CW-CRDS. The Allan deviation also indicates that the lowest stable precision for CW-CRDS needs less averaging time which is much less than the WM-DAS. After 100 s, the averages of both of the measurements show a similarly unstable deviation. This could be caused by the drifts in temperature, pressure, and cavity length for WM-DAS [29,30,31]. For CW-CRDS, this could be affected by laser wavelength drift, temperature, pressure and other factors [10,11,12].

## 6. Conclusions

This paper presents the construction of a wide range and calibration-free spectrometer based on the combination of WM-DAS and CW-CRDS methods to measure gas concentrations. The measuring range and accuracy of WM-DAS and CW-CRDS are analyzed in detail and verified by using a mixture of CO and N_2_ in various proportions and concentrations. The measurement accuracy for 3650 ppm~1.09%, 101~3650 ppm, and 4~101 ppm ranges are 5~10 ppm, 0.5~5 ppm, and ~0.5 ppm, respectively. In the range 101~3650 ppm, the baseline ringdown time, *τ*_0_, is calculated by using the absorbance measured by WM-DAS and the ringdown time with gas absorption at central frequency. The relative error between the calculated and the measured *τ*_0_ is less than 4 × 10^−4^. The gas concentration can be quantified by measuring the ringdown time at the center frequency without measuring *τ*_0_. Combining the advantages of WM-DAS and CW-CRDS, a wide and continuous range (more than five orders of magnitude), high precision, and fast (0.1 ms) spectrometer is constructed. The detection limit of this system can reach 35 ppb in 25 s. With increased precision and SNR, the range and speed of the measurements can become wider and faster, which offers a new possibility for its usability as a gas sensor in the industrial field.

## Figures and Tables

**Table 1:** CO concentration obtained by *τ*_0_, _meas._ and *τ*_0_, _calc_.

*X*_CO_ [ppm]	*τ*_meas_ [μs]	*τ*_0,meas_ [μs]	*X*_meas_ [ppm]	*τ*_0,calc_ [μs]	*X*_calc_ [ppm]	*RE* [10^−6^]
10,900	1.12	64.75	10,909.55	64.74	10,909.52	2.75
3650	3.22	64.74	3652.02		3652.02	0.00
1010	10.28	64.75	1011.14		1011.12	19.78
360	22.45	64.73	359.31		359.34	83.49
101	42.34	64.74	100.90		100.92	198.02
25	57.68	64.75	25.12		25.11	398.09

Note: *X*_meas._ denotes the concentration obtained by *τ*_0, meas_; *X*_calc_. denotes the concentration obtained by *τ*_0, meas._; *X*_CO_, standard CO concentration; and *RE* denotes the relative errors between the *X*_meas._ and *X*_calc._.
